# Pandemic Influenza A (H1N1) and Its Prevention: A Cross Sectional Study on Patients’ Knowledge, Attitude and Practice among Patients Attending Primary Health Care Clinic in Kuala Lumpur, Malaysia

**DOI:** 10.5539/gjhs.v4n2p95

**Published:** 2012-03-01

**Authors:** Latiffah A Latiff, Saadat Parhizkar, Huda Zainuddin, Goh M Chun, Mohammad Ali A Rahiman Nur Liyana N Ramli, Kerk L Yun

**Affiliations:** Department of Community Health, Faculty of Medicine and Health Sciences Universiti Putra Malaysia (UPM); Department of Public Health, Faculty of Health Yasuj University of Health and Medical Sciences, Iran; Department of Community Health, Faculty of Medicine and Health Sciences Universiti Putra Malaysia

**Keywords:** Attitude, Influenza A (H1N1), Knowledge, Malaysia, Practice, Primary health centre

## Abstract

The World Health Organization confirmed that the novel influenza A, H1N1 as a pandemic on 11 June 2009. After less than three months, 182 countries were affected by the pandemic accounting for about 150,000 infected cases and 3000 mortality. Successful H1N1 pandemic management strategies’ shaped by making changes in health behavior. The aim of this study was to document patients’ knowledge, attitudes and practices (KAP) regarding the pandemic influenza A (H1N1) and its prevention. We performed a cross-sectional study on knowledge, attitudes and practices (KAP) on preventive measures of Influenza A (H1N1) involving 322 patients attending *Klinik Kesihatan Jinjang*, a primary health care clinic in Kuala Lumpur, Malaysia from May 10 to 26, 2010 using a face to face interview with a structured pre-tested questionnaire. The majority of the respondents were females (56.8%), Malays (43.2%) aged between 18-27 years old (28.9%). There were significant association between knowledge on the complication of H1N1, effectiveness of the treatment, preventive measures of Influenza A (H1N1) and race (p<0.001) and educational level (p<0.001). There were also significant associations between attitude scores of these patients and their gender (p=0.03), and educational level (p=0.001). Practice scores related to H1N1 were found to be significantly associated with race (p<0.001) and educational level (p<0.001). The significant associations were observed between knowledge and attitude (p<0.001), knowledge and practices (p<0.001), as well as attitude and practices related to H1N1 (p<0.001). Knowledge has a crucial effect on patients’ attitude and practice particularly in a pandemic spread. So health policy makers should attempt to disseminate information about preventive measures to community in order to improve their preventive practices during pandemics.

## 1. Introduction

By April 2009, the sever cases of pneumonia were occurred in Mexico which proceeded by influenza virus and then spread to North America. The cause of that pneumonia identified and noted which it quickly evolved into a pandemic (Van *et al.*, 2009). Evidence that this strain could pass from human to human led the World Health Organization to quickly raise its pandemic alert level to Phase 6 on 11 June, 2009, it has since spread widely across the globe with substantial clinical impact (WHO, 2009). During the first wave of A (H1N1) influenza in Malaysia, 14,912 cases were reported from May 15, 2009 until Jun 4, 2010 and a total number of 88 deaths were recorded across the country in 2010 (Ministry of Health Malaysia; 2010). Effective pandemic management requires support from the population at risk for measures undertaken to mitigate the pandemic’s spread. Previous studies during the Severe Acute Respiratory Syndrome (SARS) outbreak in 2003 have shown that individual beliefs and perceptions play an important role in subsequent desired behavioral change (Tang and Wong, 2003; Lau *et al.*, 2003). Higher perception on effectiveness of measures undertaken (Lau *et al.*, 2003; Tang and Wong, 2004) and higher perceived threat of the disease led to higher rates of positive behavioral change, and better knowledge also increased the uptake of preventive measures (Leung *et al.*, 2004; Leung *et al.*, 2005). Similarly, in an anticipated H5N1 epidemic, these factors also influenced both self and community protective behavior (Lau *et al.*, 2007) During the current influenza pandemic, studies have found that the individual’s emotional status mediates behavioral response (Jones and Salathe, 2009)) and that perceived severity and susceptibility to disease and perceived effectiveness of specific behaviors resulted in the corresponding recommended behavior changes (Rubin *et al.*, 2009; Seale *et al.*, 2009). To increase positive perceptions, clear dissemination of information is vital to reduce misconceptions (Lau *et al.*, 2009). It is therefore important to perform behavioral studies in different populations to understand the determinants that influence behaviors. However, there have been few studies (Mohd Shahriman *et al.*, 2009; Zairina *et al.*, 2011) on the knowledge, attitudes and practices towards the influenza A pandemic in Malaysia. As such, there is a need to understand the factors influencing such behavioral changes to promote effective management of influenza A pandemics in this country. Therefore, this study was conducted to identify the knowledge, attitude and practice (KAP) associated with complications of H1N1, effectiveness of treatment, preventive measures and health promotion of Influenza A (H1N1) among patients attending *Klinik Kesihatan* Jinjang in Kuala Lumpur, Malaysia in 2010.

## 2. Materials and Methods

We conducted a cross-sectional study in *Klinik Kesihatan* Jinjang, Kuala Lumpur for 16 days duration (from May 10 to 26, 2010). The study protocol was approved by the Medical Research Ethics Committee of Faculty of Medicine and Health Sciences, Universiti Putra Malaysia (UPM) and permission from the director of Kuala Lumpur Federal Territory Health Department and Klinik Kesihatan Jinjang were obtained to collect the data. Participants (n=322) were randomly selected from the attendees of Klinik Kesihatan Jinjang, Kuala Lumpur according to their attendance number in the clinic and written consent was obtained from them. Eligible participants were 18 years of age or older.

### 2.1 Data Collection Tool

We developed a questionnaire to assess the knowledge, attitudes and practices (KAP) regarding pandemic influenza A which was administered to each participant by face to face interview. The questionnaire was adopted from previous studies done by researchers (Rubin *et al.*, 2009; Goodwin *et al.*, 2009), and was pilot tested among 30 patients from another clinic (Klinik Kesihatan Pantai) with similar profiles to prove its reliability. The questionnaire was consisted of 4 parts: A: demographic characteristics, B: knowledge C: attitudes and D: practices of participants using both open and close questions. The questionnaire collected information on basic demographic data on age, sex, ethnicity and education level as well as questions on knowledge, attitudes, and practices on pandemic influenza A (H1N1). Questions on knowledge were used to assess the respondent’s general knowledge on pandemic influenza A and on the recommended preventive measures. Questions on attitudes were used to assess perceptions towards pandemic influenza A and these preventive measures. Questions on practices were used to assess the actual compliance and practices of these measures. Respondents’ were asked to rate their agreement with the statements in the questionnaire, which were scored either yes/no or on a 4 point Likert scale.

A score of 1 was given for a correct response. The incorrect response and “don’t know” was classified as an incorrect response and received a “0” score. In the attitude section, responses were measured on a rating scales ranging from 1 to 4 with 1 = strongly disagree, 2 = disagree, 3 = agree, and 4 = strongly agree. The higher score on the scale of ≥3 scores indicated a greater positive attitude toward those statements. The higher practice scale of >50% of action indicated that participants were more likely to practice well with reference to those practices. Respondents who scored above the mean value were considered to have high level of knowledge, positive attitudes and good practices respectively while score below the mean value was considered having low level of knowledge, negative attitudes and poor practices.

### 2.2 Statistical Analysis

For the purpose of analysis, the individual scores were summed up to yield a total score. All statistical analyses were performed using SPSS 16 for Windows (SPSS Inc, Chicago, IL), with the level of significance set at 5%. Age was computed from the information on date of birth and date of interview. Chi-squared tests of significance were used to find the association of knowledge, attitude and behavior in relation to different age groups and different educational levels.

## 3. Results

The demographic profile of the participants is shown in Table 1. Majority of the respondents were females (56.8%), Malays (43.2%) aged between 18-27 years old (28.9%). Most of the respondents had completed secondary school education (49%) followed by pre-university level of education (28.6%). Majority of respondents (61.8%) were working either in public or private organizations in the period of study.

**Table 1:** Socio-demographic characteristics of respondents (n=322)

Social demographic characteristic		Frequency	Percentage (%)
**Age Group**	18-27	93	28.9
	28-37	66	20.5
	38-47	62	19.3
	48-57	49	15.1
	58 and above	43	13.4
	Missing data	9	2.8
**Gender**	Male	139	43.2
	Female	183	56.8
**Race**	Malay	139	43.2
	Chinese	123	38.2
	Indian	49	15.2
	Others	11	3.4
**Education Level**	No formal education or completed primary education (primary school)	72	22.4
	Completed secondary education (high school)	158	49.0
	STPM * and above	92	28.6
**Working status**	Working	199	61.8
	Not working	123	38.2

STPM

* Stand for: Sijil Tinggi Persekolahan Malaysia that meaning: Malaysia Higher School Certificate

Table 2 presents the responses of the study participants on H1N1 pandemic influenza A (H1N1) and its preventive measures. The finding showed that majority of patients (80.7%) believed that H1N1 can spread through air contact, while just one third of them agreed that touching is the other method of transmission. Majority of participants knew that covering one’s nose or mouth when sneezing (85.4%) and wearing mask (76.7) can prevent the spread of H1N1. A major portion of respondents (93.2%) believed that either washing hand with soap and water after coughing/ sneezing or avoiding crowded places are helpful in prevention of spreading H1N1. Less than Half of the respondents believed that either each H1N1 patients will experience the complications or every H1N1 infected person will die because of it.

**Table 2:** Patients’ knowledge regarding pandemic influenza A (H1N1) and its preventive measure (%)

No:	Question	Yes	No	Not Sure
**1**	H1N1 can spread through air contact	80.7	6.2	13.1
**2**	H1N1 can spread through touching	36.3	34.2	29.5
**3**	Wearing mask can prevent the spread of H1N1	76.7	21.1	2.2
**4**	Covering your nose or mouth when sneezing can prevent the spread of H1N1	85.4	12.4	2.2
**5**	Washing hand with soap and water after coughing/ sneezing can prevent the spread of H1N1	93.2	5.9	0.9
**6**	Avoiding crowded places help prevent the spread of H1N1	81.4	15.2	3.4
**7**	Every H1N1 infected person will experience complications	43.8	24.8	31.4
**8**	Every H1N1 infected person will die because of it	26.7	47.8	25.5
**9**	Quarantine at home is able to heal the infected person from H1N1	52.8	25.2	22
**10**	The purpose of quarantine at home is just to avoid the spread of H1N1 virus	81	5	14

Result (Figure 1) showed that half of the patients had good knowledge level towards pandemic influenza A (H1N1). Majority of respondents (76.4%) also had positive attitudes regarding preventive measures of influenza. Subsequently, 55.3% of the respondents showed a good practice on preventive measures of influenza (Figure 1).

**Figure:**
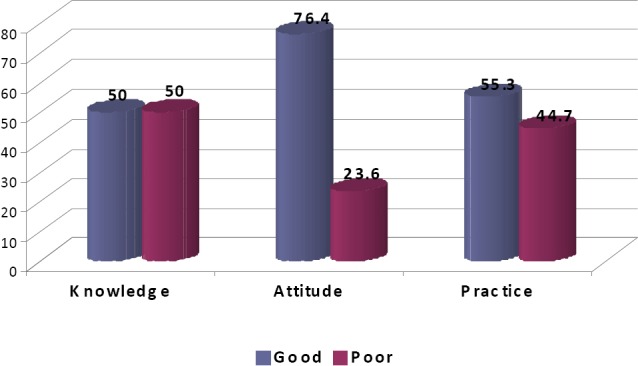
Figure 1. Knowledge, Attitude and Practice regarding preventive measures of H1N1 (%)

The level of respondents’ knowledge regarding H1N1 was not associated with different age groups, genders as well as employee status. However, there was a statistically significant associations between both race (p <0.001) and educational level (p <0.001) with knowledge regarding H1N1 (Table 3).

**Table 3:** The association between knowledge, attitudes and practices regarding Influenza A (H1N1) and socio-demographic characteristics

Socio-demographic characteristics	Knowledge Level		X²	p	Attitude Level		X²	p	Practice Level		X²	p
		
	Good	Poor			Positive	Negative			Good	Poor		
**Age Group**			5.471	0.242			3.227	0.521			6.882	0.142

**18-27**	42 (45.2%)	51 (54.8%)			21 (22.6%)	72 (77.4%)			39 (41.9%)	54 (58.1%)		
**28-37**	28 (42.4%)	38 (57.6%)			11 (16.7%)	55 (83.3%)			21 (31.8%)	45 (68.2%)		
**38-47**	37 (59.7%)	25 (40.3%)			18 (29.0%)	44 (71.0%)			33 (53.2%)	29 (46.8%)		
**48-57**	26 (53.1%)	23 (46.9%)			13 (26.5%)	36 (73.5%)			24 (49.0%)	25 (51.0%)		
**>58**	24 (55.8%)	19 (4.2%)			9 (20.9%)	34 (79.1%)			20 (46.5%)	23 (53.5%)		

**Gender**			0.114	0.736			4.712	0.030			2.395	0.122

**Male**	71 (51.1%)	68 (48.9%)			41 (29.5 %)	98 (70.5%)			69 (49.6%)	70 (50.4%)		
**Female**	90 (49.2%)	93 (50.8%)			35 (19.1%)	148 (80.9%)			75 (41.0%)	108 (59.0%)		

**Race**			23.762	<0.001			4.612	0.202			34.161	<0.001

**Malay**	51 (36.7%)	88 (63.3%)			25 (18.0%)	114 (82.0%)			41 (29.5%)	98 (70.5%)		
**Chinese**	80 (65.0%)	43 (35.0%)			33 (26.8%)	90 (73.2%)			79 (64.2%)	44 (35.8%)		
**Indian**	27 (55.1%)	22 (44.9%)			15 (30.6%)	34 (69.4%)			18 (36.7%)	31 (63.3%)		
**Others**	3 (27.3%)	8 (72.7%)			3 (27.3%)	8 (72.7%)			6 (54.5%)	5 (45.5%)		

**Educational Level**			38.463	<0.001			13.270	0.001			22.299	<0.001

**No Formal Education or Completed Primary Education**	52 (72.2%)	20 (27.8%)			26 (36.1%)	46 (63.9%)			46 (63.9%)	26 (36.1%)		
**Completed Secondary Education**	86 (54.4%)	72 (45.6%)			39 (24.75)	119 (75.3%)			73 (46.2%)	85 (53.8%)		____
**STPM or Higher**	23 (25.0%)	69 (75.0%)			11 (12.0%)	81 (88.0%)			25 (27.2%)	67 (72.8%)		____

**Working Status**			0.645	0.422			0.643	0.423			0.849	0.357

**Working**	58 (47.2%)	65 (52.8%)			32 (26.0%)	91 (74.0%)			59 (48.0%)	64 (52.0%)		
**Not Working**	103 (51.8%)	96 (48.2%)			44 (22.1%)	155 (77.9%)			85 (42.7%)	114 (57.3%)		____

The level of respondents’ attitude regarding H1N1 were not associated with respondents’ age, race and employment status, even though there were statistically significant associations between both gender (p <0.001) and educational level of patients (p <0.05) with their attitudes regarding H1N1 (Table 3).

The respondents’ practices regarding H1N1 prevention were not associated with respondents’ age, race (p <0.001) and employment status, though there were statistically significant associations between race and educational level of participants (p <0.001) with their practices regarding H1N1.

There were significant association between knowledge and attitude (p<0.001), knowledge and practices (p<0.001), (Table 4) as well as attitude and practices related to H1N1 (p<0.001).

**Table 4:** Association between respondents’ knowledge level with attitude and practice among respondents regarding Influenza A (H1N1)

Measurements		Attitude		Total	X²	P value	Practice		Total	X²	P value
	
		Negative	Positive				Bad	Good			
Knowledge Level	Low	52 (32.3%)	109 (67.7%)	161 (100%)	13.50	<0.001	95 (59.0%)	66 (41.0%)	161 (100%)	26.58	<0.001
	
	High	24 (14.9%)	137 (85.1%)	161 (100%)			49 (30.4%)	112 (69.6%)	161 (100%)		
			
Total		76 (23.6%)	246 (76.4%)	322 (100%)			144 (44.7%)	178 (55.3%)	322 (100%)

**Table 5:** Association between attitude and practice among respondents regarding Influenza A (H1N1)

Measurements		Practice		Total	X²	P value

		Bad	Good			
Attitude	Negative	55 (72.4%)	21 (27.6%)	76 (100%)	30.760	<0.001

	Positive	89 (36.2%)	157 (63.8%)	246 (100%)				
Total		144 (44.7%)	178 (55.3%)	322 (100%)				

## 4. Discussion

The results of the present survey depicts a range of knowledge, attitudes and self reported behavioural patterns concerning H1N1 among a sample of adult population in a primary health care clinic in Malaysia. This study investigated the levels of knowledge, attitudes and practices regarding H1N1 and should provide scientific support to assist health sector authorities’ in developing strategies and health education campaigns to prevent transmission of H1N1. Guidelines and recommendations have been developed to prevent and control the spread of H1N1 during pandemic threat (WHO, 2009). Successful containment or control of pandemic influenza will rely on early recognition of sustained human-to-human transmission which requires a system for outbreak detection, rapid data collection, analysis, assessment and timely reporting.

The current study revealed significant relationships between race, educational level and knowledge level which were consistent with the study conducted by Sukprasert and his colleagues in Thailand (2009). On the other hand, the result showed no significant associations between some demographic characteristics (e.g. age, gender and working status) and knowledge level that consistently was similar to other study done among rural population in Jempol, Malaysia (Mohd Shahriman *et al.*, 2009). Similarly, the results indicated that gender, and educational level significantly associated with knowledge level that was also highlighted in the study done by Marshall (2009) in South Australia where women were found to be more likely than men to report high concerns about the threat of pandemic influenza A (H1N1). Janahi and his colleagues (2011) in Bahrain also reported that higher education and older age have positively correlated with an increase in the general knowledge about the virus and the disease. Both race and educational level were found to have been associated significantly with practices regarding H1N1 prevention.

Both practices and knowledge level towards preventive measures on H1N1 were only affected by different types of races and education levels. The reasons may be because the campaigns were done in Malay or English language, which most of the other races do not understand, thus, they do not get the knowledge needed to practice the preventive measures recommended by the Ministry of Health.

The triad of knowledge, attitudes and practices in combination governs all aspects of life in human societies, and all three pillars together make up the dynamic system of life itself. Therefore, they were linked all together in a way so that any increase in knowledge, changes in attitudes towards prevention of Influenza A H1N1 as well as changes in the kinds of practices that were followed regarding prevention of H1N1.

There was significant association between knowledge and attitude (p=0.001). This was consistent with the result of study done in Jempol, situated in Negeri Sembilan, about 200km away from Kuala Lumpur (Mohd Shahriman *et al.*, 2009). There was also significant association between knowledge and practices. Meanwhile, in a study done among Thai medical students, Wisedjinda (2009) reported that there was no significant correlation between knowledge and practices. The other researcher (Sukprasert *et al.*, 2009) also stated that there was no significant association between knowledge and practices. There was also significant association between attitude and practices (p=0.001). This concurred with the findings of Wisedjinda (2009) and Zairina *et al.*, (2011) confirming a significant association between attitude and practices. This study has some limitation as the study population was only confined to adults and limited in one health centre which its generalizability is questionable. Although results in this study are useful in pandemic preparedness, the community response may change over time as compared to the time of study. This study was a cross sectional survey and may not have been able to assess the true association between knowledge and practices. Thus future study with different study design should be considered for validation.

## 5. Conclusion

Knowledge has significant influence on attitude and practices in a pandemic spread of Influenza A (H1N1), and personal experience influences individual’s practices and behaviors. It is suggested that education measurement is one of the important keys of pandemic containment strategies. Efforts should be targeted at inculcating relevant knowledge and educating the general population to improve practices in the current pandemic, as well as for future epidemics. Therefore, in the future, educational programs should focus on improving good practices for containing community transmission of pandemic influenza A (H1N1).

## References

[ref1] Goodwin R, Haque S, Neto F (2009). Initial psychological responses to Influenza A, H1N1 (“Swine flu”). BMC Infect Dis.

[ref2] Janahi E, Awadh M, Awadh S (2011). Public knowledge, risk perception, attitudes and practices in relation to the swine flu pandemic: A cross sectional questionnaire-based survey in Bahrain. International Journal of Collaborative Research on Internal Medicine & Public Health.

[ref3] Jones J. H, Salathe M (2009). Early assessment of anxiety and behavioural response to novel swine origin influenza A (H1N1). PLoS One.

[ref4] Lau J. T, Griffiths S, Choi K. C (2009). Widespread public misconception in the early phase of the H1N1 influenza epidemic. J Infect.

[ref5] Lau J. T, Kim J. H, Tsui H. Y (2007). Anticipated and current preventive behaviors in response to an anticipated human-to-human H5N1 epidemic in the Hong Kong Chinese general population. BMC Infect Dis.

[ref6] Lau J. T. F, Yang X, Tsui H (2003). Monitoring community responses to the SARS epidemic in Hong Kong: from day 10 to day 62. J Epidemiol Community Health.

[ref7] Leung G. M, Ho L. M, Chan S. K (2005). Longitudinal assessment of community psychobehavioral responses during and after the 2003 outbreak of severe acute respiratory syndrome in Hong Kong. Clin Infect Dis.

[ref8] Leung G. M, Quah S, Ho L. M (2004). A tale of two cities: community psychobehavioral surveillance in Hong Kong and Singapore during the severe acute respiratory syndrome epidemic. Infect Control Hosp Epidemiol.

[ref9] Marshall H, Ryan P, Roberton D (2009). Pandemic Influenza and Community Preparedness. American Journal of Public Health.

[ref10] (2010). Ministry of Health Malaysia. Public Health Crisis Management: The Malaysian Scenario. Disease Control Division, 17 June 2010.

[ref11] Mohd Shahriman A. F, Sin Y. K (2009). A cross sectional study on the socio-demographic factors in knowledge, attitude, and practice (KAP) on influenza A H1N1 among residents of Felda Palong, Jempol, Negeri Sembilan. MD dissertation. University Putra Malaysia.

[ref12] Rubin G. J, Amlot R, Page L (2009). Public Perceptions, Anxiety, and Behaviour Change in Relation to the Swine Flu Outbreak: Cross Sectional Telephone Survey. BMJ.

[ref13] Seale H, McLaws M. L, Heywood A. E (2009). The community’s attitude towards swine flu and pandemic influenza. Med J Aust.

[ref14] Sharifirad G. h, Yarmohammadi P, Morovati Sharifabad M. A (2011). The Status of Preventive Behaviors regarding Influenza (A) H1N1. Pandemic Based on Protection Motivation Theory among Female High School Students in Isfahan in 2009. Health System Research.

[ref15] Sukprasert A, Chotruangprasert T, Rujiputtanakul C (2009). Knowledge, attitude and practice of pandemic H1N1 influenza prevention in Thailand. http://202.28.191.133/ramaInter/sites/default/files/0CM/PDF/1_ApichayaSukprasert__Poster_7Oct2009%281%29%281%29.pdf.

[ref16] Tang C. S. K, Wong C. Y (2003). An outbreak of the severe acute respiratory syndrome: predictors of health behaviours and effect of community prevention measures in Hong Kong, China. Am J Public Health.

[ref17] Tang C. S. K, Wong C. Y (2004). Factors influencing the wearing of facemasks to prevent the severe acute respiratory syndrome among Chinese in Hong Kong. Prev Med.

[ref18] Van D, McLaws M. L, Crimmins J (2010). University life and pandemic influenza: Attitudes and intended behaviour of staff and students towards pandemic (H1N1). BMC Public Health.

[ref19] Wisedjinda L, Hengriprasopchoke S, Karucote A (2009). Knowledge, attitude, and practices concerning influenza A H1N1 2009 among Thai medical students. http://www.ra.mahidol.ac.th/ramaInter/sites/default/files/0CM/PDF/2_Napas_H1N1%20poster_6Oct2009%281%29.pdf.

[ref20] (2009). World Health Organization. Global surveillance during an influenza pandemic. Version 1. Updated April 2009.

[ref21] Zairina A. R, Nooriah M. S, Yunus A. M (2011). Knowledge And Practices Towards Influenza A (H1N1) Among Adults in Three Residential Areas in Tampin Negeri Sembilan: A Cross Sectional Survey. Med J Malaysia.

